# Inactivated Mycobacterial Vaccine Nebulized Inhalation: A Effective Therapy for the Prevention and Treatment of Respiratory Diseases?

**DOI:** 10.1111/crj.70101

**Published:** 2025-07-01

**Authors:** Xiaohong Jiang, Qixiang Sun, Yujia Huang, Yuetian Deng, Chaoqian Li

**Affiliations:** ^1^ Department of Geriatric Respiratory Medicine The First Affiliated Hospital of Guangxi Medical University Nanning Guangxi China; ^2^ Department of Respiratory and Critical Care Medicine Affiliated Hospital of Youjiang Medical University for Nationalities Baise Guangxi China; ^3^ Department of Endocrinology Medicine, Cadre Ward Guangxi Medical University Kaiyuan Langdong Hospital Nanning Guangxi China; ^4^ Department of Emergency The First Affiliated Hospital of Guangxi Medical University Nanning Guangxi China

**Keywords:** BCG, inactivated, mycobacterial vaccine, *Mycobacterium phlei*, *Mycobacterium vaccae*, nebulized inhalation

## Abstract

Nebulized inhalation therapy is an important method in the prevention and treatment of respiratory diseases, and inactivated mycobacterial vaccine nebulized inhalation has received a wide attention recently, but the roles and mechanisms are still not fully understood. A literature search showed there is a strong scientific rationale and evidence that nebulized inhalation of inactivated mycobacterial vaccine is effective in the prevention and treatment of respiratory diseases. Clinically available mycobacterial vaccines include *Mycobacterium phlei* (*M. phlei*), BCG, and *Mycobacterium vaccae* (*M. vaccae*). Nebulized inhalation of inactivated mycobacterial vaccine, especially *M. vaccae*, has been used in the prevention and treatment of respiratory diseases, such as asthma, respiratory syncytial virus (RSV), coronavirus disease 2019 (COVID‐19), and sepsis. It acts on the respiratory tract directly, thus stimulating the body to produce an immune response, enhance respiratory immunity, and achieve prevention and treatment effects. Nebulized inhalation of inactivated mycobacterial vaccine will be an effective therapy in the prevention and treatment of respiratory diseases.

## Graphical abstract

Nebulized inhalation of inactivated *Mycobacterial vaccine* will be an effective therapy for the prevention and treatment of respiratory diseases.

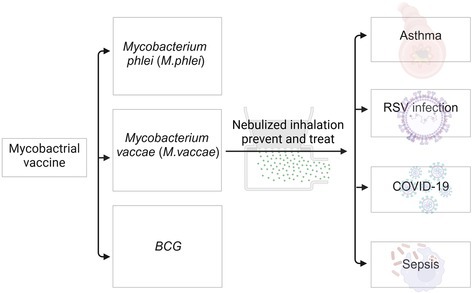

## 1 Nebulized Inhalation Therapy History

Nebulized inhalation therapy is a direct administration method with the respiratory tract and lung as the target organs. It offers rapid onset of action and is associated with minimal systemic exposure, therefore reducing the risk of adverse effects [1]. So, it has been used as an important treatment method for respiratory diseases. It uses physical means, such as ultrasonic vibration, compressed gas dynamics, or mesh vibrations, to convert the liquid drug into aerosol form to form fine particles that can be inhaled by the patient through the natural breathing process. These particles can penetrate deep into the respiratory tract and reach the alveoli to achieve a highly effective local therapeutic effect, improving the efficacy of the drug while reducing systemic side effects [2]. It is especially suitable for the management of acute attacks; compared with oral or intravenous administration, nebulization can reduce systemic drug exposure and reduce the risk of adverse reactions, and it is suitable for a wide range of people, from infants to elderly patients.

Nebulized inhalation medications for the treatment of respiratory diseases have a long history [3], and the nebulizer development has also gone through a long process [4]. Nebulized inhalation therapy for asthma and other complaints origins date back 4000 years [5]. It is a very old method of drug delivery [3]. In 1928, antiseptic inhalations were advocated for the treatment of tuberculosis [4]; in 1946, penicillin nebulization was used in bronchopulmonary diseases such as bronchiectasis and infection [6].

## 2 Inactivated Mycobacterial Vaccines and Their Immunological Basis

*Mycobacterium* is intertwined with human life; among approximately 190 species in this genus, mainly *Bacillus Calmette–Guérin* (BCG) and *Mycobacterium tuberculosis* (*M*tb) are studied intensively [7]. To date, clinically available mycobacterial vaccines include *Mycobacterium phlei* (*M. phlei*), BCG, and *Mycobacterium vaccae* (*M. vaccae*).

*M. phlei*, a gram‐positive acid‐fast mycobacterium from the *Actinomycetes Mycobacteriaceae* family, was first discovered by German scientists in 1889; it has no spores or flagella and has a few 1.0‐ to 2.0‐μm‐long rod‐shaped branches. *M. phlei* has the cell wall structure of both gram‐positive (G^+^) and gram‐negative bacteria (G^−^), and it is found widely in soil, plants, and drinking water [8]. Its cell structure is quite similar to that of prokaryotic bacteria except for the unique cell wall [9]. Normally, *M. phlei* is nonpathogenic to humans and other animals practically, but its cell wall and other components have strong immune regulation, growth promotion, anti‐infection, and other effects after inactivation. A large number of experiments have demonstrated that *M. phlei* bacteria, cell wall, DNA, unmethylated CpG, polysaccharides, and so on have antitumor, antiradiation, and other effects and are very potential immunomodulators, which are widely used in clinical practice; it can treat many chronic diseases and tumors, such as recurrent respiratory tract infections, bronchial asthma, drug‐resistant tuberculosis, and malignant tumors (like as non–small‐cell lung cancer, gastric cancer, and bladder cancer).

BCG vaccine, an attenuated strain of *Mycobacterium bovis* , is obtained by serial passage, whereas in 1908, *M. bovis* was isolated by Albert Calmette and Camille Guérin firstly [10]. BCG is widely used as a vaccine [11], it still was the gold‐standard treatment therapy for nonmuscle‐invasive bladder cancer for more than 40 years [12]. On the other hand, it not only can be used for the prevention of tuberculosis but also has a nonspecific protective effect on humans called “trained immunity” mediated by innate immune cells such as monocytes, macrophages, and natural killer cells [13]. There still has been research improving that BCG vaccination was unable to cause a long‐term reinforcement of Th1 response in asthmatic children, although it could avoid the increased Th2 response observed in control patients [14]. BCG is also used to decrease recurrent respiratory tract infections and acute chronic bronchitis [15]. However, the side effects of BCG, such as allergic reactions and fever, have affected its application; scientists have tried to find alternatives that have no side effects and good immune effects, so people have focused their research on *M. phlei* that are not pathogenic, but unfortunately, *M. phlei is* not produced now.

*M. vaccae*, a nonpathogenic species belonging to the same genus as *M. tuberculosis* , is commonly found in soil and water and was first described in 1964 [16]. It is a preparation with bidirectional immunomodulatory effect made by high‐temperature inactivation and purification of *M. bovis* by high temperature. It is available in injectable form in China (Anhui Longkema Biological Pharmaceutical Co., China) and is approved in China as an immunotherapeutic agent [17]. It is often used in the treatment of respiratory diseases such as asthma and tuberculosis [18, 19, 20] and also used in the treatment of lung cancer [21], and it is the only immunotherapeutic agent that is recommended by WHO in the Tuberculosis Strategic Development Plan of 1991 [17].

To date, the mycobacterial vaccines like *M. phlei*, BCG, and *M. vaccae* are widely used in respiratory diseases and bladder cancer, mainly through intramuscular injection. From literature research, we found that nebulized inhalation of inactivated mycobacterial vaccines has a strong treatment and prevention effects on respiratory diseases.

## 3 Nebulized Inhalation of Inactivated Mycobacterial Vaccine and Respiratory Diseases

The details of this method are as follows: nebulized with 22.5‐μg *M. vaccae* (Anhui Longkema Biological Pharmaceutical Co., Anhui, China) mixed with 10‐mL phosphate‐buffered saline (PBS) once daily for five consecutive days [22].

### 3.1 Nebulized Inhalation of Inactivated Mycobacterial Vaccine and Bronchial Asthma

Bronchial asthma is a serious global health problem, affecting approximately 300 million people around the world and causing around 1000 deaths per day [23]. It is a heterogeneous disease, usually characterized by chronic airway inflammation, airway hyperresponsiveness, and airway hypersecretion, defined by the recurrent attacks of respiratory symptoms, such as cough, wheeze, shortness of breath, and chest tightness. The symptoms vary over time and in intensity, together with variable expiratory airflow limitation. The main pathogenesis of asthma includes hyperresponsiveness, airway inflammation, and high mucus secretion. Professor Li's research team from China has investigated the effect of nebulized inactivated mycobacterial vaccine on asthma from the basic and clinical perspectives. They found it can significantly alleviate airway inflammation, airway hypersecretion, and improve the clinical symptoms of asthma in both asthmatic patients and mice.

Back in 2012, Zhang et al. found that the nebulized inhalation of *M. phlei* can alleviate airway inflammation in asthmatic mice; this is attributed to its immunomodulatory effect on regulating IL‐4, IL‐10, and IFN‐γ secretion [24], also correlated with modulating γδT cell function. It showed that the expression of IL‐10^+^γδT cells, IFN‐γ^+^γδT cells, and Vγ4 mRNA were significantly increased after nebulized inhalation of inactivated *M. phlei* [25]. It is known that γδT cells are important modulators of airway hyperresponsiveness and allergic inflammation. Researchers demonstrated that Vγ1^+^γδT cells can increase eosinophilic airway inflammation and airway hyperresponsiveness, while Vγ4^+^γδT cells reduce airway hyperresponsiveness [26]. Subsequently, they made discoveries in clinical research and demonstrated that nebulized inhalation of inactivated *M. phlei* , to some extent, improved asthmatic symptoms, reduced the need for rescue medication, and reduced acute exacerbation in adult asthmatic patients. It played the same role as inhaled Seretide treatment in reducing airway hyperresponsiveness [27]. Immediately afterwards, Ming et al. showed the same effect in moderately asthmatic children [28]. Through animal research, they also proved further mechanisms of nebulized inhalation of inactivated *M. phlei* in asthmatic mice. It can significantly reduce the IL‐5 and IL‐13 levels in lung and IgE level in BALF [29], modulate the balance of CD4^+^CD25^+^ regulatory T cells and Th17 cells [30], and reduce the IL‐17^+^γδT cell‐mediated immune response of asthma in mice [31]. These studies proved that *M. phlei* nebulized inhalation may be accepted as an alternative method in asthmatic treatment with less risk of adverse reactions.

**FIGURE 1:**
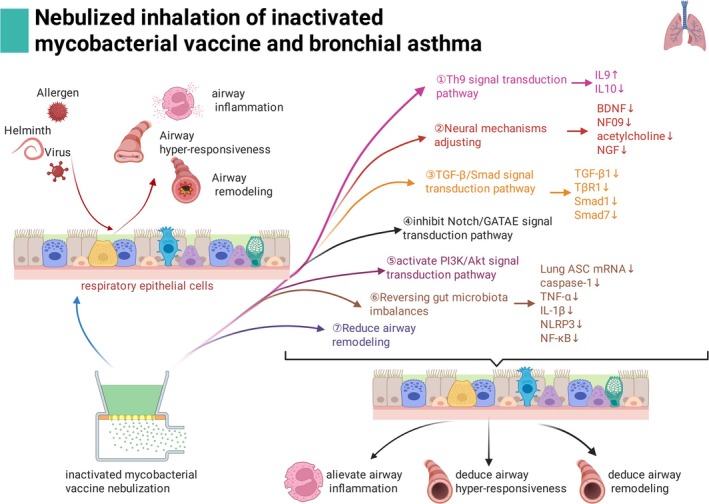
The mechanisms of nebulized inhalation of inactivated mycobacterial vaccine and bronchial asthma.

On the other hand, the other inactivated mycobacterial vaccine, called *M. vaccae*, attracted the attention of the researchers gradually. There was a research that showed that *M. vaccae* intratracheal administration exerted a lasting ameliorating effect on airway histopathologic features of asthmatic mice [32]. Vaccination with *M. vaccae* in OVA‐sensitized pregnant BALB/c mice can prevent Th2 immune responses by enhancing IFN‐γ secretion and lowering IL‐5 levels during pregnancy, and this effect persisted during the postnatal period in offspring [33] and so on. So, in 2016, Li et al. first used nebulized inhalation therapy of *M. vaccae* in the treatment and prevention of asthma in mice; the results demonstrated that both the airway inflammation and airway hyperresponsiveness in asthmatic mice were alleviated and reduced after being treated with nebulized inhalation of *M. vaccae*. The mechanisms could involve the Th9 signal transduction pathway, *M. vaccae* ‐mediated effects on the induction of IL‐9 secretion, and suppression of IL‐10 secretion from γδT cells [22]. From then on, this research team began a series of studies on the relationships between nebulized inhalation therapy of *M. vaccae* and asthma. Their research results showed that nebulized inhalation of *M. vaccae* could indeed alleviate airway inflammation, reduce airway hyperresponsiveness, and airway remodeling in asthmatic mice. The mechanisms include (Figure 1): (1) Th9 signal transduction pathway. It has been described as above. (2) Neural mechanisms adjusting. It was proven that the expression of BDNF, NF09, acetylcholine, and the level of NGF mRNA were decreased in the asthmatic mice after treatment with *M. vaccae* [34]. (3) TGF‐β/Smad signal transduction pathway [35]. It showed that after nebulized inhalation of *M. vaccae*, the expression of TGF‐β1, TβR1, Smad1, and Smad7 of the TGF‐β/Smad signal transduction pathway was deregulated in asthmatic mice. (4) Notch/GATA3 signal transduction pathway. The result demonstrated that the *M. vaccae* ‐primed γδT cells can alleviate asthmatic symptoms in mice by reversing lung Th2 polarization and inhibiting the Notch/GATA3 signaling transduction pathway [36]. (5) PI3K/Akt signal transduction pathway. The newest research from Xiao et al. proved that nebulized inhalation of *M. vaccae* can decrease eosinophil counts; alleviate airway inflammation, mucus secretion, and airway remodeling in asthmatic mice through autophagy inhibition; and reduce the levels of IgE, IL‐5, IL‐13, and TNF‐α by inhibiting autophagy; it can also suppress autophagy in IL‐13‐stimulated BEAS‐2B cells. *M. vaccae* nebulization may protect against asthma by activating the PI3K/Akt signaling pathway [37]. (6) Reversing gut microbiota imbalances. We know that host immunity can influence the composition of the gut microbiota and affect disease progression consequently. It is proven that this method can alleviate airway inflammation and hyperresponsiveness in asthmatic mice by reversing imbalances in gut microbiota [38]. Xiao et al. also proved that *M. vaccae* nebulized inhalation can inhibit the mRNA expression of lung ASC, caspase‐1, TNF‐α, and IL‐1β, and also inhibit the expression of lung NLRP3 and NF‐κB protein during allergen sensitization or challenge [39]. (7) Reduce airway remodeling. Xiao et al. demonstrated that the expression of IL‐1β, TNF‐α, NF‐κB, and WISP1 mRNA in the pulmonary tissue of asthmatic mice was downregulated, while β‐catenin, WISP1, and Wnt1 protein were inhibited, and glycogen synthase kinase‐3 beta (GSK‐3β) was upregulated after nebulized inhalation of *M. vaccae* . Nebulized inhalation of *M. vaccae* can reduce airway remodeling in asthmatic mice [39]. These mechanistic insights are expected to pave the way for therapeutic strategies for asthma.

### 3.2 Nebulized Inhalation of Inactivated Mycobacterial Vaccine and Respiratory Syncytial Virus (RSV)

RSV is the leading cause of acute lower respiratory tract infection, especially in young children [40]. Former research demonstrated that *M. vaccae* nebulization can protect against allergic asthma, as RSV infection and bronchial asthma are closely related [41]. We further hypothesized whether nebulized inhalation of *M. vaccae* can protect against pulmonary RSV infection. Therefore, we investigated the effect of *M. vaccae* nebulization on RSV infection in BALB/c mice. One week before the RSV infection mouse model was established, the mice in the RSV infection group were all nebulized with *M. vaccae* once a day for five consecutive days. The results showed that after 1 week *of M. vaccae* intervention, in the RSV infection group, the mice's airway inflammation was alleviated, the pulmonary mRNA levels of RSV, NF09, acetylcholine, and EGFR expression were decreased considerably, whereas the TLR7 and TLR8 mRNA levels were increased significantly. It further proved the effect of *M. vaccae* nebulization on RSV infection; the mechanism may be the effect of *M. vaccae* on the regulation of neurotransmitters and expression of TLR7, TLR8, and EGFR [42].

### 3.3 Nebulized Inhalation of Inactivated Mycobacterial Vaccine and Coronavirus Disease 2019 (COVID‐19)

During the COVID‐19 pandemic, the research team of Professor Li also explored the efficacy, safety, and effect of inactivated mycobacterial vaccine inhalation on the treatment of COVID‐19. They conducted a randomized, double‐blind, and placebo‐controlled clinical trial; a total of 31 adult patients with moderate COVID‐19 were included. They were randomly divided into two groups. The primary outcome was the time interval from admission to viral RNA negative conversion (in this study, the oropharyngeal swabs were used), the secondary outcomes included chest computed tomography (CT), mortality, the length of hospital stay, and complications during the treatment. Patients were followed up to 4 weeks after discharge (viral RNA were reexamined, chest CT, etc.). The results showed that *M. vaccae* nebulized inhalation shortened the time interval from admission to viral RNA negative conversion, which might be beneficial to the prevention and treatment of COVID‐19 [43].

### 3.4 Nebulized Inhalation of Inactivated Mycobacterial Vaccine and Sepsis

Sepsis often causes acute lung injury and has a high clinical mortality rate; it is thought to be related to a variety of inflammatory mediators, but the pathogenesis remains unclear. *M. phlei* cell wall, polysaccharides, and other components have immunomodulatory functions. As an inactivated mycobacterium, can *M. phlei* alleviate lung injury in sepsis? Professor Gu from China demonstrated that *M. vaccae* nebulized inhalation can reduce lung damage by reducing the levels of sepsis‐related inflammatory factors and pathway proteins. It has certain clinical significance.

### 3.5 Nebulized Inhalation of Inactivated Mycobacterial Vaccine and Other Diseases

To date, our literature search results have not found reports of nebulized inhalation of inactivated mycobacterial vaccines for other diseases.

## 4 Summary and Prospects

Nebulized inhalation of inactivated mycobacterial vaccine has the advantages of noninvasiveness, simple operation, and safety; it is suitable for the majority of patients. This method can prevent and treat a variety of respiratory diseases and has a wide range of applications. Immunomodulation may be the main mechanism. The most studied currently was the *M. vaccae* nebulized inhalation effect on respiratory diseases. In bronchial asthmatic mice, it can alleviate airway inflammation, deduce hyperresponsiveness, reduce airway modeling, and so on. In RSV infection mice, it can reduce the RSV mRNA level. In COVID‐19 patients, it can shorten the time interval from admission to viral RNA negative conversion.

This method, nebulized inhalation of inactivated mycobacterial vaccine, has been granted patents in China and has been applied in clinical practice. With further research, nebulized inhalation of inactivated mycobacterial vaccine may become an important means for the prevention and treatment of respiratory diseases and will provide new ideas and methods for the prevention and treatment of respiratory diseases.

However, despite its many advantages, there are still some challenges and problems. For example, the specific mechanism of this method still needs to be further studied; at the same time, more clinical trials are needed to verify the differences in efficacy between different populations and different disease types.

## Author Contributions

**Xiaohong Jiang:** writing – original draft. **Qixiang Sun:** writing – original draft. **Yujia Huang:** writing – review and editing. **Yuetian Deng:** writing – review and editing. **Chaoqian Li:** writing – review and editing, funding acquisition. All authors read and approved the final manuscript.

## Conflicts of Interest

The authors declare no conflicts of interest.

## Data Availability

The data that support the findings of this study are available on request from the corresponding author. The data are not publicly available due to privacy or ethical restrictions.
